# The Fatigue Assessment Scale as a simple and reliable tool in systemic lupus erythematosus: a cross-sectional study

**DOI:** 10.1186/s13075-019-1864-4

**Published:** 2019-03-25

**Authors:** Alice Horisberger, Delphine Courvoisier, Camillo Ribi

**Affiliations:** 1 Division of Immunology and Allergy, Department of Medicine, Centre Hospitalier Universitaire Vaudois, CHUV BH09-627, CH-1011 Lausanne, Switzerland; 2 Division of Rheumatology, Department of Internal Medicine Specialties, University Hospitals of Geneva, Geneva, Switzerland

**Keywords:** Systemic lupus erythematosus, Fatigue Assessment Scale, Disease activity

## Abstract

**Background:**

The vast majority of patients with systemic lupus erythematosus (SLE) complain about fatigue. They also report fatigue as one of their most debilitating symptoms. Yet, in clinical practice, fatigue is only rarely assessed and remains poorly understood. The purpose of this study is to validate the Fatigue Assessment Scale (FAS) and assess the impact of disease activity on fatigue in SLE.

**Methods:**

A cross-sectional single-center study of patients was included in the Swiss SLE Cohort Study. The FAS and the Short Form 36 (SF-36) were administered to SLE patients and controls with primary Sjogren’s syndrome (pSS) and healthy volunteers (HV) attending our clinic. Disease activity in SLE was captured at the same time as patient-reported outcomes using the SLE Disease Activity Index score with the Safety of Estrogens in SLE National Assessment modification (SELENA-SLEDAI) and the physician’s global assessment. We explored the internal consistency, reproducibility, construct validity, and convergence of the FAS, in comparison to the vitality subscale (VT) of the SF-36. We examined the association of FAS with demographics, disease type, SLE disease activity, and clinical features.

**Results:**

Of the 73 SLE subjects, 89% were women and 77% were Caucasians. The median age was 43 years, and 23 (32%) patients had active SLE. Demographics in pSS and HV were similar. Within the SLE group, FAS displayed good internal consistency (Cronbach’s alpha = 0.93), unidimensionality, and test-retest reliability (ICC = 0.90). FAS and VT correlated well. The total FAS was highest in active SLE and pSS and higher in non-active SLE compared to HV.

**Conclusion:**

The FAS is a promising tool to measure fatigue in SLE. Patients with SLE display a significantly higher level of fatigue than HV, which is even more pronounced in active disease and comparable to fatigue levels measured in pSS.

## Key messages

- This study is the first to validate the Fatigue Assessment Scale (FAS) as a reliable and simple tool to assess fatigue in systemic lupus erythematosus.
- Fatigue assessed by FAS correlates with global disease activity in systemic lupus erythematosus.
- Fatigue levels in active systemic lupus erythematosus are comparable to those in matched controls with Sjogren’s syndrome.

## Background

Systemic lupus erythematosus (SLE) is a chronic autoimmune disease with a wide spectrum of clinical and biological manifestations. The multiple dysfunctions in the innate and adaptive immune system that ultimately lead to autoimmunity and inflammation are thought to be triggered by various environmental factors in genetically susceptible individuals [1]. The vast majority of SLE patients complain about fatigue [2, 3] and report it as one of the most debilitating disease features [4, 5]. Whether fatigue reflects SLE disease activity is still a matter of debate [6]. Lupus fatigue has important repercussions on daily activities and is associated with poor quality of life (QoL) [7]. By using a qualitative interview tool, Sterling et al. highlighted its negative impact on emotional, cognitive, professional, and social status [8]. Given its major role on patient’s morbidity, fatigue is an essential aspect to consider in SLE management. Yet, fatigue remains poorly understood by both health care providers and the patient’s entourage [9].

Multiple instruments for assessing fatigue and various definitions are available, without one standing out for its simplicity and reliability. Thus, fatigue is difficult to evaluate in daily practice and to standardize across studies for research purposes. The Fatigue Assessment Scale (FAS) is a simple 10-item self-reported questionnaire designed by Michielson et al. to assess fatigue in the general population and validated subsequently in the sarcoidosis setting [10, 11]. The FAS is derived from a pool of 40 items selected in four previous valid questionnaires: the Fatigue Scale, the Checklist Individual Strength, the Emotional Exhaustion subscale of the Dutch version of the Maslach Burnout Inventory, and the Energy and Fatigue subscale of the World Health Organization Quality of Life assessment instrument. The face validity was studied through a semantical analysis in order to guide the selection of items. The FAS is reportedly a unidimensional scale measuring fatigue independently from depression [12]. It has proven to be a reliable and valid tool as well as sensitive to change in sarcoidosis patients [13]. Owing to its good psychometric properties in this specific disease, this instrument was then used in a placebo-controlled randomized clinical trial evaluating the effect of *N*-acetylcysteine on fatigue in SLE [14, 15]. However, the reliability and validity of FAS in SLE patients have yet to be demonstrated. In this study, the primary objective was to study the construct, the convergent validity, and test-retest correlation of the FAS in SLE patients. Secondary objectives were to compare the fatigue score between SLE patients, non-SLE patients, and healthy volunteers; to measure perceived fatigue in SLE patients with active and inactive disease; and to determine whether other factors contribute to fatigue.

## Methods

### Study population

Participants were aged ≥ 18 years old and attended the Centre Hospitalier Universitaire Vaudois (CHUV) in Lausanne between June 2015 and July 2016. All were included in the Swiss Systemic Lupus Erythematosus Cohort Study (SSCS) [16, 17]. All SLE patients fulfilled the revised American College of Rheumatology (ACR) criteria and/or the Systemic Lupus International Collaborating Clinics (SLICC) criteria [18, 19]. Control groups consisted of patients with primary Sjogren syndrome (pSS) meeting the 2002 American-European Classification Criteria [20] and age- and gender-matched healthy volunteers (HV). Patients with completed FAS and SF-36 forms were retained for the cross-sectional study. Patients with pSS were asked to participate in the cohort study during their regular clinical follow-up. The HV were recruited by public notice in the CHUV and were evaluated medically to confirm the good health and absence of autoimmune disease or immunomodulatory treatment. The protocol was approved by the Canton Vaud ethical committee. All participants gave their written informed consent, and the study was carried out in compliance with the Helsinki Declaration.

#### Data collection and instruments

Data on patient’s age, sex, ethnicity, educational status, tobacco use, body mass index (BMI), disease duration since diagnosis, activity and damage, and treatment modalities were collected during the medical visit. Disease activity in SLE was assessed by the SLE Disease Activity Index with the Safety of Estrogens in Lupus Erythematosus National Assessment modification (SELENA-SLEDAI) [21]. This score is based on 24 clinical and biological items, which reflect disease activity within the past month. Disease activity was also evaluated using the Physician’s Global Assessment score (PGA) with a 4-point-Likert-scale ranging from 0 (inactive) to 3 (very active disease) [21]. Patients with a SELENA-SLEDAI ≥ 4 and a PGA ≥ 1 were considered to have active disease [22]. Damage was assessed in SLE with the SLICC/ACR Damage Index (SDI) [23]. Disease-modifying treatment (DMARD) at study visit and in the 4 weeks before was classified into three groups: systemic glucocorticoids, antimalarials, and immunosuppressants. Data on health-related quality of life (HRQoL) and fatigue were assessed with the Short Form 36 (SF36) and the FAS in all participants. The SF36 is a widely used health survey form measuring 8 dimensions of QoL; each of them ranges from 0 to 100 with lower scores reflecting poorer health. The SF-36 vitality subscale (VT-SF36) is used for convergent validity of fatigue questionnaires [24]. The mental health subscale of the SF-36 (MH-SF36) was used for the discriminant validity. The FAS comprises 10 questions with answers varying from never to always on a 5-point-scale. The total FAS score ranges from 10 to 50, increasing proportionally to fatigue. Both questionnaires were completed by participants during the study visit. A sample of 30 SLE patients was asked to complete the FAS 2 weeks after the first assessment. These patients were reminded by text messages to complete and send back the questionnaire after 2 weeks with a pre-stamped envelope. Eligibility for this test-retest study was a completed form, and the absence of important intercurring events reported by the patient that would influence the state of fatigue during the test-retest period. FAS, SF-36, demographical data, and clinical data were collected in the two control groups of pSS patients and HV.

#### Statistical procedure

Descriptive statistics were presented as absolute count and percent for qualitative data and as median and interquartile range (IQR) for quantitative. Difference between groups and correlations were evaluated using non-parametric tests. The psychometric properties of the FAS were evaluated using the following methods. Cronbach’s alpha was calculated to measure the reliability of the tool by looking at inter-item consistency. This measure is generally considered satisfactory if the alpha value is above 0.7 for group-level analysis but a value above 0.9 is desirable for individual patients in clinical application [25]. A factor analysis was performed using the principal component analysis (PCA) based on Kaiser criteria (eigenvalue > 1) and visual inspection of the scree plot. A PCA was used with the FAS and the MH-SF36 in order to examine the divergent validity (construct validity) of the FAS. Factor analysis extraction was presented with an oblimin rotation. A coefficient factor above 0.3 was considered significant. As there were slightly less than 5 (4.87) patients per item for this measure, we confirmed the results on a larger sample using SLE, pSS, and HV. The convergent validity was examined with a Spearman’s rho correlation between FAS and VT-SF36. The test-retest reliability was measured using intraclass correlations (ICC). A linear regression model was used for multivariable analysis, with total FAS score as dependent variable and disease activity and corticoid use as independent variables. *P* values *<* 0.05 were considered significant. All statistics were performed on IBM SPSS statistics 24 (IBM Corp Armonk, NY) and GraphPad Prism 7 (GraphPad Software, La Jolla, CA, USA).

## Results

### Group characteristics

Seventy-three patients with SLE, 23 patients with pSS, and 23 healthy volunteers were included (Table 1). There was no statistical difference in demographic features between groups. SLE subjects were predominantly women (89%), of Caucasian origin (77%) and had a median age of 43 years at assessment. Median disease duration from diagnosis was 7 (3–15) years in patients with SLE compared to 1 (0–2) in those with pSS (*p* <  0.001). Patients with SLE were more frequently treated with systemic corticosteroids and immunosuppressant drugs compared to those with pSS. Among SLE patients treated with immunosuppressants, four had received cyclophosphamide in the month preceding the study. Within the SLE group, 23 (32%) had active disease (SELENA-SLEDAI ≥ 4 and PGA ≥ 1). Table 2 compares the characteristics of SLE patients according to their disease activity.

**Table 1:** Patient’s and volunteer’s characteristics

	All SLE (*n* = 73)	pSS (*n* = 23)	HV (*n* = 23)	*p* value^§^
Age, median (IQR) years	43 (34–57)	44 (33–58)	34 (22–49)	0.44
Female sex (%)	65 (89)	21 (91)	20 (87)	0.89
Caucasian, no. (%)	56 (77)	19 (83)	20 (87)	0.53
Higher education*, no./total (%)	30/59 (51)	10/22 (45)	10/23 (43)	0.80
Active smoker, no. (%)	17 (23)	4 (17)	8 (35)	0.37
Body mass index, median (IQR)	23 (20–28)	25 (22–28)	22 (20–24)	0.48
Use of psychiatric medication^£^, no. (%)	14 (19)	8 (34)	3 (13)	0.16
Time from diagnosis, median (IQR) years	7 (3–15)	1 (0–2)	–	< 0.01
Immunomodulators past month, no. (%)	64 (88)	16 (70)	–	0.04
Antimalarials, no. (%)	52 (71)	14 (61)	–	0.35
Systemic corticosteroids, no. (%)	39 (53)	5 (22)	–	0.01
Immunosuppressants, no (%)	35 (48)	5 (22)	–	0.03

*SLE* systemic lupus erythematosus, *pSS* primary Sjogren syndrome, *HV* healthy volunteers, *IQR* interquartile range

*Defined as higher professional or technical school or university degree

^£^Defined as any antidepressants, antipsychotics, and anxiolytics/hypnotics

^§^*p* value < 0.05 is considered significant

**Table 2:** Clinical characteristics of systemic lupus erythematosus patients according to global disease activity (*n* = 73)

	Inactive SLE (*n* = 50)	Active SLE (*n* = 23)	*p* value^§^
Age, median (IQR) years	46 (35–59)	39 (30–52)	0.20
Female sex (%)	44 (88)	21 (91)	0.68
Caucasian, no. (%)	38 (76)	18 (78)	0.83
Higher education*, no./total (%)	20/39 (51)	10/20 (50)	0.93
Active smoker, no. (%)	9 (18)	8 (35)	0.12
Body mass index, median (IQR)	24 (20–29)	22 (19–24)	0.35
Use of psychiatric medication^£^, no. (%)	9 (18)	5 (22)	0.75
Disease duration, median (IQR) years	7 (3–15)	6 (3–15)	0.42
History of lupus nephritis, no (%)	19 (38)	6 (26)	0.31
History of neurolupus, no (%)	6 (12)	2 (7)	0.67
SELENA-SLEDAI, median (IQR) score	2 (0–2)	6 (4–10)	< 0.001
PGA score, median (IQR) score	0 (0–0)	1 (1–2)	< 0.001
Immunomodulators past month, no. (%)	44 (88)	20 (87)	0.90
Antimalarials, no. (%)	37 (74)	15 (65)	0.44
Systemic corticosteroids, no. (%)	26 (52)	13 (57)	0.72
Immunosuppressants, no. (%)	25 (50)	10 (43)	0.60

*IQR* interquartile range, *SLE* systemic lupus erythematosus

*Active disease is defined as a Systemic Lupus Erythematosus Disease Activity Index score with the Safety of Estrogens in Lupus Erythematosus National Assessment modification (SELENA-SLEDAI) ≥ 4 and a Physician’s Global Assessment score (PGA) ≥ 1

^£^Defined as any antidepressants, antipsychotics, and anxiolytics/hypnotics

^§^*p* value < 0.05 is considered significant

### Psychometric analysis of FAS

**Fig. 1:**
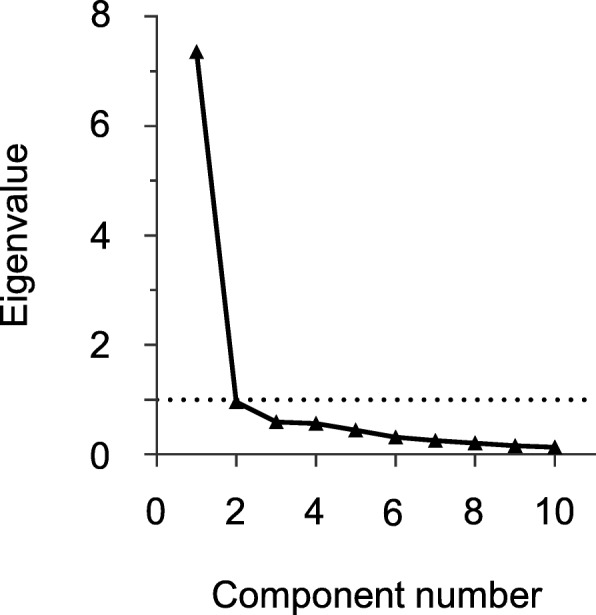
Scree plot of the Fatigue Assessment Scale (FAS) and its items

The internal consistency of the FAS was measured at 0.93 (Cronbach’s alpha coefficient), and none of the items would have improved the internal consistency if removed. Principal component analysis favored a one-component solution, confirmed by visual inspection of the scree plot (Fig. 1). The factor analysis of FAS items extracted a unique factor explaining 64% of the variance with the items loading between 0.68 (item 3) and 0.88 (item 5).

Of the 73 SLE subjects, 30 (41%) had a test-retest assessment. Seven patients were excluded because of a self-reported significant event between the first and the second FAS assessment. There was no difference in age, sex, ethnicity, and disease activity between the whole SLE sample and the test-retest subjects (data not shown). The test-retest correlation was good with an ICC of 0.90 (95%CI 0.80–0.95, *p* <  0.001) for a 2-week interval. The FAS correlated strongly with the VT-SF36 subscale (*r*_*s*_ = 0.85, *p* <  0.001). The FAS correlation with the MH-SF36 was *r*_*s*_ = 0.65 (*p* < 0.001). The PCA on the combined pool of the FAS and the MH-SF36 items favored a two-component solution (eigenvalue factor I, 8.4; factor II, 1.9; percentage explained variance, 69%). One statement of the ten FAS items (FAS-7) loaded higher on the mental health scale. All other items loaded higher on the fatigue factor. All items of the MH-SF36 loaded higher on the mental health factor (Table 3). These results were similar using a larger sample including SLE, pSS, and HV (data not shown) except for the item FAS-7 showing a higher loading on the fatigue factor (0.48) than on the mental factor (0.42).

**Table 3:** Factor loading of the FAS and the MH-SF36 obtained in 73 patients with SLE in a two-factor solution

Items	Factor 1 - fatigue	Factor 2 - mental health
I am bothered by fatigue (FAS-1)	0.94^†^	0.10
I get tired very quickly (FAS-2)	0.94^†^	0.08
I do not do much during the day (FAS-3)	0.75^†^	0.08
I have enough energy for everyday life (FAS-4)	− 0.74^†^	− 0.13
Physically, I feel exhausted (FAS-5)	0.89^†^	0.01
I have problems starting things (FAS-6)	0.62^†^	− 0.32^†^
I have problems thinking clearly (FAS-7)	0.37^†^	− 0.51^†^
I feel no desire to do anything (FAS-8)	0.65^†^	− 0.25
Mentally, I feel exhausted (FAS-9)	0.62^†^	− 0.25
When I am doing something, I can concentrate quite well (FAS-10)	− 0.77^†^	− 0.20
Have you been a very nervous person? (SF09B)	0.18	0.97^†^
Have you felt so down in the dumps that nothing could cheer you up? (SF09C)	− 0.05	0.87^†^
Have you felt calm and peaceful? (SF09D)	0.20	− 0.67^†^
Have you felt downhearted and low? (SF09F)	0.02	0.91^†^
Have you been a happy person? (SF09H)	0.17	− 0.68^†^

*FAS* Fatigue Assessment Scale, *MH-SF36* mental health component of the Short Form 36 health survey questionnaire

^†^A coefficient factor above 0.3 was considered significant

### Comparison of fatigue levels between groups

The FAS score was significantly increased in both SLE and pSS compared to healthy subjects. Median (IQR) FAS was 23 (17–32) in SLE, 27 (20–34) in pSS, and 16 (14–18) in HV (*p* = 0.001). These fatigue findings were confirmed using the VT-SF36 subscale (median VT score [IQR] = 45 [23–58] in SLE, 35 [15–50] in pSS, and 70 [50–75] in HV, *p* = 0.001). There was a good correlation between the FAS and the VT-SF36 score among all groups of participants (Table 4). There was a lesser but significant correlation between FAS and the other subscales of the SF36 in both SLE and pSS. In contrast, HV showed modest to the non-significant correlation between FAS and SF-36 subscales other than VT. In the three groups of participants, no correlation was found between the total FAS score and demographic features such as age, sex, ethnicity, educational status, and BMI (data not shown). Tobacco use was weakly associated with a higher degree of fatigue in the pSS group (*p* = 0.024). The use of psychiatric medication was associated with higher fatigue levels in both SLE (*p* = 0.002) and pSS (*p* = 0.047) patients, but not in HV.

**Table 4:** Correlation coefficient between the FAS and the SF-36 subscores

	FAS		
SF-36 subscores	SLE (*n* = 73)	pSS (*n* = 23)	HV (*n* = 23)
Physical functioning	− 0.58*	− 0.73*	− 0.25
Role physical	− 0.59*	− 0.58*	− 0.32
Role emotional	− 0.60*	− 0.64*	0.00
Bodily pain	− 0.68*	− 0.67*	0.23
General health	− 0.71*	− 0.70*	− 0.50*
Vitality	− 0.85*	− 0.78*	0.85*
Social functioning	− 0.67*	− 0.47*	0.52*
Mental health	− 0.65*	− 0.60*	− 0.50*

*FAS* Fatigue Assessment Scale, *SF-36* Short Form 36, *SLE* systemic lupus erythematosus, *pSS* primary Sjogren’s syndrome, *HV* healthy volunteers

*Correlations are significant at *p* value < 0.05

### Association of fatigue and disease activity in SLE patients

**Fig. 2:**
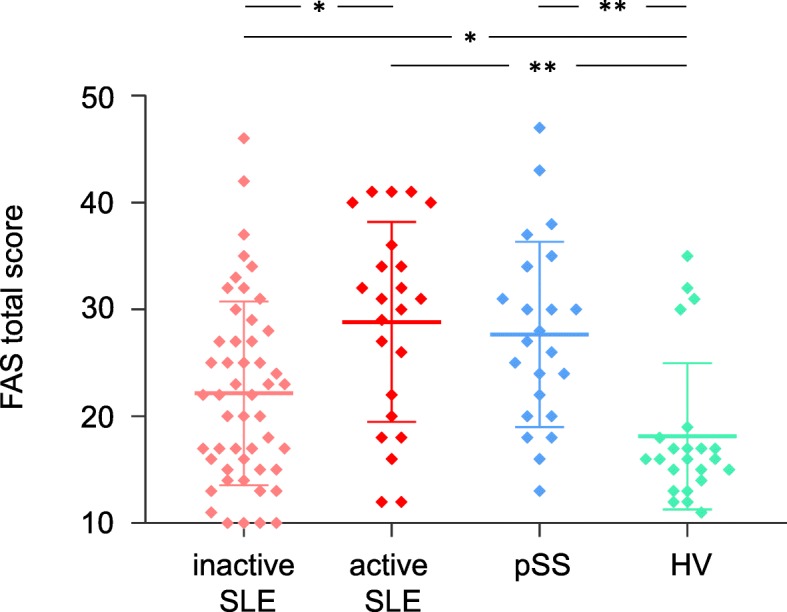
Fatigue Assessment Scale (FAS) scores in 73 patients with systemic lupus erythematosus (SLE) according to disease activity and controls with primary Sjogren’s syndrome or in good health. SLE systemic lupus erythematosus, FAS Fatigue Assessment Scales (ranges from 10 – no fatigue to 50 – extreme fatigue), pSS primary Sjogren’s syndrome, Healthy healthy volunteers. Plots represent the individual values (diamonds), the median score, and the IQR for each group. *p < 0.05; **< 0.001

The median (IQR) FAS score was 31 (20–36) in patients with active SLE, compared to 22 (15–27) in those with inactive disease (*p* = 0.005). In healthy volunteers, the FAS score was slightly lower than in patients with inactive SLE (*p* = 0.05). In contrast, patients with active disease presented with a significant higher level of fatigue than HV (*p* < 0.001) (Fig. 2). No association was found between fatigue and other clinical parameters such as the time elapsed from diagnosis to assessment, number of ACR criteria fulfilled at inclusion, presence of renal involvement at any time during disease course, presence of auto-antibodies (anti-dsDNA and anti-SSA/Ro) at study visit, and damage accrual (SDI score). There was no difference in levels of fatigue between SLE patients with or without a history of renal disease (median [IQR] FAS was 24 [17–32] and 23 [16–31], respectively). Fatigue was higher in the patients treated with corticosteroids (*r* = 0.27, *p* = 0.022). There was no difference when comparing fatigue with the use of other DMARDs. In a linear regression model, after adjusting for corticosteroids and psychiatric medication use at visit, active disease remained significantly associated with fatigue so that FAS increased on average by 6.2 points (95%CI 2.2–10.3, *p* = 0.003) for each additional point in disease activity.

## Discussion

To the best of our knowledge, ours is the first study that validates the FAS as a simple and reliable tool to assess fatigue in SLE. The FAS measures fatigue by means of 10 items including two reverse questions. This fatigue scale was previously shown to be useful and valid in the general population, in the working population, and in sarcoidosis patients. Fatigue is a prominent feature of SLE, a disease known for its wide range of symptoms. Brain fog is a common complaint in SLE patients, which refers to periods of impaired cognition [26] without any signs of neurolupus. Indeed, only a very few of the patients in this study had overt neurological disease. Fatigue is also a major complaint in other autoimmune diseases such as pSS. The thin line between cognitive dysfunction and depression in SLE and pSS makes fatigue assessment a particular challenge in this population. Our study shows that the FAS displays solid psychometric abilities, with an excellent internal consistency and test-retest reliability. Its convergent validity is supported by the good correlation with the VT-SF36. Concerning discriminant validity, it is revealed that fatigue and mental disorders such as depression are related but distinct constructs.

This cross-sectional study also shows that fatigue measured by FAS is significantly increased in both SLE and pSS patients compared to healthy controls. Several authors reported similar results for chronic inflammatory diseases, including SLE and pSS [27, 28]. In previous studies, the prevalence of fatigue in SLE subjects varied from 76 to 90% [3, 29, 30]. By comparing FAS to the SF-36 subscales, we confirm the relationship between fatigue and various aspects of HRQoL, such as perceived mental health, emotional state, bodily pain, and social functioning in both SLE and pSS. We found that fatigue levels were higher in patients using psychiatric medication. Whether fatigue is the cause or the consequence of mental health issues and prescribed psychiatric medication in these patients remains to be established. SLE patients in our study differed from those with pSS in terms of disease duration from diagnosis, which was significantly shorter for pSS. On the other hand, pSS has a more insidious disease course than SLE. Diagnosis of pSS is often delayed by years and the duration of symptoms difficult to establish. This may explain why the time elapsed since pSS and SLE diagnosis in our study had no influence on the measured fatigue levels and reflects our clinical impression of long-standing fatigue in most patients suffering from these conditions. Moreover, we show that patients with active SLE have significantly more fatigue than healthy controls, whereas this difference is much less pronounced in those with inactive SLE. The association between fatigue and disease activity in SLE is controversial. Some authors reported a lack of association between disease activity and the Fatigue Severity Score [30–32]. Others, however, were able to show that fatigue increases with SLE activity, although to various degrees [3, 14, 33]. In the present study using two global scores (SELENA-SLEDAI and PGA) to ascertain SLE disease activity, we demonstrate a clear positive correlation with fatigue. Interestingly, patients with active SLE displayed a similar degree of fatigue than those with pSS. This observation amplifies the need for further investigation of immune factors that could contribute to fatigue in both active pSS and SLE. Recently, Petri et al. demonstrated a significant decrease in fatigue and disease activity in SLE patients treated with blisibimod, a selective inhibitor of B cell activating factor, in a phase 2b study. The authors found a weak correlation between disease activity and fatigue and concluded that this symptom appears to be closely related to immune dysfunction [33, 34]. Others have found that the use of IL-6 blocking agents has a favorable impact on fatigue in SLE [35]. These findings could be explained by the impact of pro-inflammatory cytokines on the central nervous systemic with induction of illness behavior, expressed in symptoms such as fatigue and anhedonia [36]. The relief of fatigue after the use of various biological agents supports a relationship between fatigue and inflammation due to ongoing disease activity. We did not find any association of fatigue with traditional markers, such as auto-antibodies. This underlines the need for further studies to assess fatigue levels in relation to circulating cytokines and novel biomarkers in autoimmune disease.

Our study has some limitations. We did not assess additional factors potentially contributing to fatigue, such as sleep disorders, depression, and physical activity. Our study is cross-sectional and does not allow the determination of the sensitivity to change. Longitudinal studies with FAS are needed to assess variations in fatigue, and its causes and its impact on health-related quality of life and working capacity both in SLE and pSS.

## Conclusion

In conclusion, this study validates the FAS as a simple and reliable tool to assess fatigue in patients with SLE and shows a close and positive correlation of fatigue with disease activity, independently from corticosteroid use and psychiatric medication.

## Abbreviations

BMI: Body mass index
CHUV: Centre Hospitalier Universitaire Vaudois
DMARD: Disease-modifying treatment
FAS: Fatigue Assessment Scale
HRQoL: Health-related quality of life
HV: Healthy volunteers
ICC: Intraclass correlation coefficient
IQR: Interquartile range
MH-SF36: Mental health subscale of the SF-36
PCA: Principal component analysis
PGA: Physician’s Global Assessment score
pSS: Primary Sjogren’s syndrome
QoL: Quality of life
SDI: SLICC/ACR Damage Index
SELENA-SLEDAI: SLE Disease Activity Index score with the Safety of Estrogens in SLE National Assessment modification
SF-36: Short Form 36
SLE: Systemic lupus erythematosus
SLICC: Systemic Lupus International Collaborating Clinics
SSCS: Swiss Systemic Lupus Erythematosus Cohort Study
VT: Vitality subscale
